# Supplementary material for: Co-Production at Work: The Process of Breaking Up Sitting Time to Improve Cardiovascular Health. A Pilot Study
Source: Int J Environ Res Public Health. 2021 Dec 30;19(1):361. doi: 10.3390/ijerph19010361 (PMC8744924; doi:10.3390/ijerph19010361)
Supplement: Supplementary file 1 [file ijerph-19-00361-s001.zip › ijerph-1472722-supplementary.pdf]

Supplementary Material

Table S1*: Summary of the co-production participatory process and its associated objectives*

Development Stage	*Timeline*	*Participatory Process:*	*Objectives*	*Tasks/key questions*
1. Needs analysis	June 2020	**Online Questionnaire**	1. To gather an understanding of current physical activity and sedentary behaviour habits.<br>2. To gather an understanding of the opinions and views of a workplace physical activity intervention focused at breaking up sitting time<br>3. To attain preliminary opinion of the acceptability of breaking up sitting time with physical activity during the working day<br>4. To gather opinions on different interventions (including resistance and walking interventions) in the workplace	• What are your physical activity habits at the workplace?<br>• How long do you typically spend sitting across a working day?<br>• What workplace physical activity provision have you have access to at work?<br>• What are your thoughts on physical activity being incorporated into your workplace?<br>• What type of intervention?<br>• What are your perceptions about breaking up sitting time with physical activity during the working day?
2. Eligibility & Intervention Framework	(November 2020)	**Online Focus Group**	1. Highlight the issues of prolonged sitting<br>2. To obtain further thoughts from the stakeholders regarding the acceptability for the intervention, from an employee and management level<br>3. To gain perceptions on the preliminary	• Introduction – (i) summarise questionnaire findings and (ii) provide an illustrative summary of the research around prolonged sitting<br>• What needs to happen to enable

			thoughts of what the intervention should look like	change to occur in the working day?<br>• What does this intervention look like in practice/workplace setting?<br>• What issues/challenges are there for implementation?
3. Intervention framework	(February 2021)	Online Focus Group	1. To address the structural and functional components of the physical activity intervention e.g. what the physical activity looks like, how the participants will be supported during the intervention.<br>2. Note and address perceived barriers of the intervention	• Prior to the meeting, a preliminary intervention framework will be designed based on the needs analysis and eligibility stages and from consultation of the research team.<br>• Share the framework with the group to discuss issues of delivery and feasibility, and to further inform the intervention through refinements to the proposed model.<br>• To gain further feedback for the refined framework from the initial development group
4.Intervention Piloting	(March 2021)		1. To practice run the intervention with the development group to put the design to practice.	• Delivery of the intervention with a sample (n=5) of the development group.<br>• Participants were asked to note challenges and motivators during

				the intervention as they arise.
5. 'Follow-Up' intervention development	(March 2021)	**Online Focus Group**	1. Primary objective: to summarise the outcome of the process, check for consensus and gather further comments following the piloting on the intervention.<br>2. Secondary objective: Establish actionable refinements to improve the intervention.	• Identify challenges and motivators<br>• Make necessary changes from the pilot.
